# Successful treatment of hemophagocytic syndrome in a patient with T cell lymphoma, EBV infection, and bone marrow necrosis: A case report

**DOI:** 10.1097/MD.0000000000028943

**Published:** 2022-03-04

**Authors:** Lingling Xu, Xianqi Liu, Yan Wang, Yanming Wang, Xiaoxia Chu, Liming Chen

**Affiliations:** a Department of Hematology, Yantai Yuhuangding Hospital Affilliated to Qingdao University, Yantai, China; b Department of Burn and Plastic Surgery, Yantai Yeda Hospital Affilliated to Binzhou Medical College, Yantai, China.

**Keywords:** bone marrow necrosis, Epstein–Barr virus, hemophagocytic syndrome, rituximab, T cell lymphoma

## Abstract

**Rationale:**

Hemophagocytic syndrome (HPS) is associated with a high mortality rate, and Epstein–Barr virus infection and hematological malignancies, especially T/natural killer cell lymphomas, are the most common causes; however, due to the complexity of clinical manifestations, the diagnosis is usually delayed. There are few reports of lymphoma-associated HPS (LAPS) in combination with bone marrow necrosis, and there is still no standard treatment for LAPS.

**Patient concerns:**

A 64-year-old man developed a fever, mild jaundice, fatigue, and bone pain. Positron emission tomography and bone marrow biopsy with immunohistochemistry were performed.

**Diagnosis:**

Imaging analysis and bone marrow examinations were compatible with HPS, T-cell lymphoma, and bone marrow necrosis.

**Interventions:**

The patient received combination therapy of rituximab and Cyclophosphamide, epirubicin, vincristine, glucocorticoid, etoposide.

**Outcomes:**

The patient achieved complete remission and a disease-free survival of 52 months.

**Lessons:**

HPS and its potential diseases should be diagnosed and treated as soon as possible. Clinicians should be aware of the presence of lymphoma in patients with HPS. Rituximab plays an important role in the prognosis of HPS, particularly Epstein–Barr virus positivity. Cyclophosphamide, epirubicin, vincristine, glucocorticoid remains an effective regimen for the treatment of T-cell LAPS. This study provides a better understanding of the diagnosis and treatment of LAPS.

## 1 Introduction

Hemophagocytic syndrome (HPS), also known as hemophagocytic lymphohistiocytosis is a rare but life-threatening hyperinflammatory syndrome caused by the uncontrolled proliferation of activated T lymphocytes and macrophages, which secrete high levels of inflammatory cytokines. It can cause multiorgan damage and has poor outcomes and high mortality. It usually manifests as persistent high fever, cytopenia, liver function damage, hepatosplenomegaly coagulopathy, hyperferritinemia, and hemophagocytosis in the bone marrow or other organs.^[1,2]^

There are 2 recognized forms of HPS: primary HPS, caused by an inherited genetic abnormality, and secondary HPS, often triggered by underlying conditions, including infections, malignancies, autoimmune diseases, and acquired immune deficiency.^[3]^ Epstein–Barr virus (EBV) infection and hematological malignancies, particularly T phenotype non-Hodgkin lymphomas, are the most common causes of HPS.^[4]^

Bone marrow necrosis (BMN) secondary to lymphoma-associated HPS (LAPS) is rarely reported and there is no specific treatment regimen. Here, we describe the case of a 64-year-old man who presented with EBV infection, BMN, and HPS, and was subsequently diagnosed with T-cell lymphoma. The patient was treated with rituximab combined with chemotherapy and remission was achieved.

## 2 Case report

A 64-year-old man was admitted on March 3, 2017 with a 1-month recurrent fever, fatigue, cough, mild jaundice, and 1-week new-onset ostealgia. His past medical history included diabetes mellitus and radical subtotal gastrectomy for gastric cancer 8 years ago, and the tumor cells were confined to the submucosa without lymph node or distant metastasis. He denied night sweats, weight loss, chronic liver disease, or a history of alcohol consumption or drug abuse.

**Figure 1:**
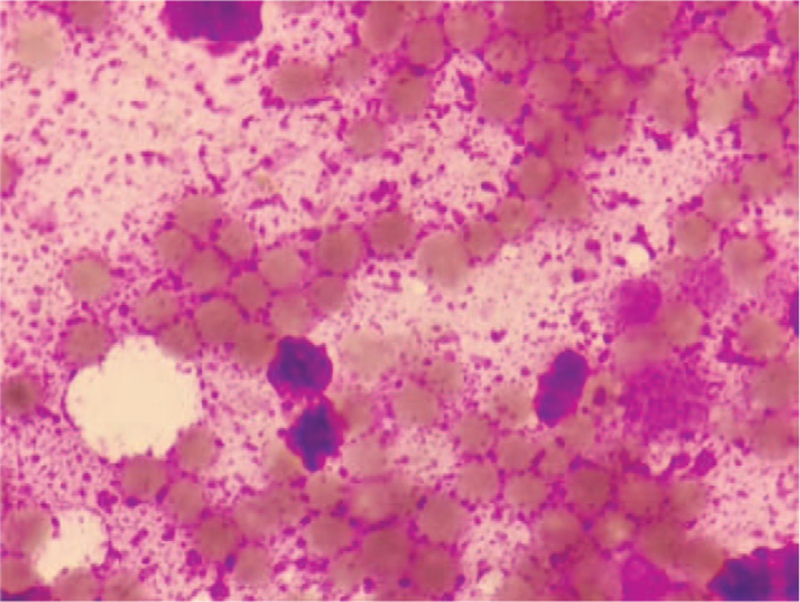
Bone marrow smears (Wright stain, ×1000) showed necrotic cells with no cell membrane in a background of amorphous proteinaceous material.

On physical examination, he was febrile (39.9°C), with pallor, jaundice of the skin and sclera, lower extremity edema, and severe backache. The spleen was located 3 cm below the left costal margin, and there was no palpable lymphadenopathy. Laboratory examinations revealed pancytopenia: white blood cell count, 1.73 × 10^9^/L; absolute neutrophil count, 0.89 × 10^9^/L; hemoglobin level, 78 g/L; and platelet (PLT) count, 26 × 10^9^/L. Liver function tests showed: the following alanine aminotransferase was 137 U/L; aspartate aminotransferase was 254 U/L; and lactate dehydrogenase, 2090 U/L. The coagulation function test showed a low level of fibrinogen at 0.89 g/L. Blood cultures were performed more than 3 times and yielded negative results. His C-reactive protein level was 52.7 mg/L. Serum triglyceride and ferroprotein level were elevated at 2.76 mmol/L and 8890 ng/mL, respectively. The viral workup was negative for influenza A, cytomegalovirus, human immunodeficiency virus, respiratory syncytial virus, and hepatitis B and C. EBV was positive with a high replication rate of 1.56 × 10^6^ copies/mL. A positron emission tomography (PET) scan was performed, which revealed intense uptake of 18F-fluorodeoxyglucose in the spleen, thoracic and lumbar vertebrae, spine, pelvis, and bilateral femur. Bone marrow aspiration and biopsy were promptly performed; however, there was no trace of cells in the smears, and a large amount of amorphous extracellular eosinophilic proteinaceous material was observed (Fig. 1). Flow cytometric immunophenotypic analysis of the bone marrow specimen was negative for leukemia and monoclonal B or T cells. Furthermore, the karyotype analysis was also normal.

**Figure 2:**
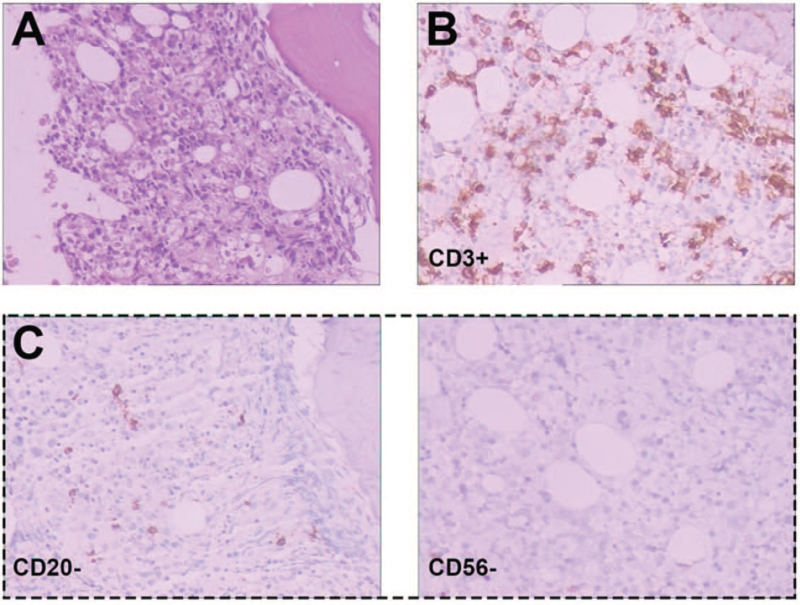
Histopathologic features. (A) BM biopsy (hematoxylin and eosin ×400) shows patchy interstitial infiltration of abnormal cells. By immunohistochemistry, the tumor cells are positive for CD3, CD8 (B) and negative for CD20, CD5, CD4, CD56, EBER (C). BM = bone marrow, EBER = epstein-barr encoded RNAs.

The patient was transferred to the intensive care unit because of hypoxic respiratory failure and received mechanical ventilation on day 2. He received supportive care, including empirical antibiotics, PLT and fibrinogen transfusion, liver protectants, and antiviral therapy. Laboratory results showed that coagulation function deteriorated rapidly and plasma fibrinogen decreased to 0.75 g/L. Serum triglyceride and ferroprotein levels high at 4.55 mmol/L and 37,257ng/mL, respectively. Soluble cluste of differentiation (CD)25 detection is up to 48,186.93 pg/mL and natural killer (NK) cell activity decreased to 12.53%. The peripheral blood cell count progressively decreased, and there was no improvement in liver function. Based on the clinical presentations and laboratory results, he was diagnosed with HPS and treated with etoposide (vp-16) 75 mg/m^2^ intravenous (IV) twice weekly and dexamethasone 20 mg IV daily. Rituximab (375 mg/ m^2^) was also administered for HPS. He was transferred to the general ward on day 5, and his clinical symptoms improved. Subsequent bone marrow biopsy and T-cell receptor gene rearrangement confirmed T-cell lymphoma, and immunohistochemical analysis revealed the expression of CD20, paired box domain 5, CD3, CD5, CD4, CD8, CD56, CD10, B Cell Leukemia-6, multiple myeloma oncogene 1, and epstein-barr encoded RNAs (Fig. 2). Following the diagnosis of LAPS, the patient was treated with a mini-Cyclophosphamide, epirubicin, vincristine, glucocorticoid (CHOP) regimen consisting of cyclophosphamide (375 mg/m^2^ on day 1), liposomal doxorubicin (10 mg/m^2^ on day 1), vindesine (3 mg/m^2^ on day 1), dexamethasone (10 mg on days 1–5), etoposide (50 mg/m^2^ on days 1, 3, and 5), and sufficient IV fluid to prevent tumor lysis syndrome. Owing to a progressive increase in liver enzymes, the dose of etoposide was reduced. Within 3 weeks after the initial chemotherapy, the patient's symptoms such as fever, cytopenia, hepatic function damage, hypofibrinogenemia, and hyperferroproteinemia improved significantly (Table 1), and EBV titers were below the threshold for reliable quantification. The patient achieved partial remission after 4 cycles of the standard Cyclophosphamide, epirubicin, vincristine, glucocorticoid, etoposide (CHOPE) regimen and complete remission after 8 cycles of PET-computerized tomography (CT), and the bone marrow biopsy showed no lymphoma cell involvement. The patient has survived for 52 months.

**Table 1:** Laboratory data during chemotherapy in patient.

	WBC	HGB	PLT	ALT	AST	FIB	FER	TG
Time point	(×10^9^L^−1^)	(g/L)	(×10^9^L^−1^)	(U/L)	(U/L)	(g/L)	(ng/mL)	(mmol/L)
Prechemo	1.73	94	26	222	243	0.89	34,371	3.6
Postchemo (d)								
3	1.35	66	19	200	215	1.03	37,257	4.55
6	0.55	67	14	166	395	1.28	36,196	2.35
9	0.59	68	6	238	230	2.64	7929	1.88
15	0.71	71	16	59	17	3.70	2560	0.79
21	18.4	77	67	20	13	3.39	3175	1.9
28	12.3	78	481	10	14	4.80	1836	1.41

ALT = alanine aminotransferase, AST = aspartate aminotransferase, Chemo = chemotherapy, FER = ferritin, FIB = fibrinogen, HGB = hemoglobin, PLT = platelet, TG = triglycerides, WBC = white blood cell count.

## 3 Discussion

HPS is characterized by high levels of inflammatory cytokines, including interferon-γ, interleukin-6 (IL-6), IL-18, IL-12, and tumor necrosis factor-α (TNF-α), which are secreted by activated T cells, excessive proliferous macrophages, and monocytes.^[1]^ The cytokine storm is responsible for the clinical manifestations and laboratory data observed in multiorgan damage, as seen in HPS. IL-6, IL-1, and TNF-α are responsible for fever, whereas interferon-γ and TNF-α contribute to hypertriglyceridemia by inhibiting lipoprotein lipase and stimulating triglyceride synthesis. These cytokines also inhibit normal hematopoiesis-inducing cytopenia.^[5]^

Primary HPS usually involves genetic mutations, such as PRF1, UNC13D, STX11, SH2D1A, RAB27A or STXBP2 and typically presents during childhood. Allogeneic hematopoietic stem cell transplantation is the only treatment that can cure primary HPS. Secondary HPS can occur at any age, and malignancy, especially LAPS, is very common.^[6]^ Han et al^[7]^ reported 29 patients with LAPS and found that 24 of these cases were associated with T-cell or NK/T-cell lymphomas and demonstrated a poor prognosis and a high mortality rate. The median survival time for these patients was 36 days. However, the exact pathogenesis of LAPS remains to be elucidated. Tumor cells secrete cytokines and activate cytotoxic T cells, which can trigger HPS.

EBV is the most common viral pathogen associated with HPS and is strongly linked to non-Hodgkin lymphoma, particularly T-cell and NK cell lymphoma.^[4]^ It has been reported that EBV plays an important role in the development of LAPS as well as EBV-associated HPS without lymphoma. EBV can infect T cells and activate T lymphocytes to secrete proinflammatory cytokines such as TNF-α and induce macrophage activation. EBV-infected T lymphocytes express latent membrane protein-1, which is a member of the TNF receptor family, and can activate nuclear factor kappa-light-chain-enhancer of activated B cells. Activated nuclear factor kappa-light-chain-enhancer of activated B cells may facilitate resistance of EBV-infected T lymphocytes to TNF-α-induced apoptosis. Therefore, EBV-positive HPS is more likely to relapse and have poor outcomes.^[8,9]^ Therefore, timely diagnosis and appropriate therapy are 2 important factors for improving the prognosis of HPS or LAPS.

The currently accepted treatment for HPS was published by the hemophagocytic lymphohistiocytosis study group of the Histiocyte Society in 2004.^[6]^ Five out of the following 8 criteria must be fulfilled: fever (>38°C); splenomegaly; cytopenia affecting at least 2 lineages of the following: hemoglobin <90 g/L, PLTs <100 × 10^9^/L, neutrophils <1 × 10^9^/L; hypertriglyceridemia (>3 mmol/L) and/or hypofibrinogenemia (<1.50 g/L); ferritin >500 ng/mL; hemophagocytosis in the bone marrow, spleen, lymph node, or liver; low or absent NK cell activity; and soluble CD25 >2400 μ/mL. However, none of the above are specific for HPS, and some of the test results can also be seen in other diseases; thus, the diagnosis of HPS is usually delayed due to the wide variety of clinical presentations and nonspecific findings. Some patients with LAPS have no common signs of lymphoma, such as lymph node enlargement; therefore, it is difficult to diagnose at an early stage. In this situation, bone marrow biopsy and PET-CT scanning are important diagnostic methods. In the present case, the patient presented with fever, cytopenia, low fibrinogen levels, hyperferritinemia, hypertriglyceridemia, splenomegaly, and markedly elevated serum EBV-deoxyribonucleic acid level, which led to the diagnosis of HPS. In addition, the patient exhibited liver function impairment with high levels of alanine aminotransferase and aspartate aminotransferase, and it was confirmed that the liver is one of the most frequently involved organs in HPS.^[10]^ The PET-CT scan showed extensive bone damage and an increased standard uptake value; therefore, we suspected a possible malignancy, especially lymphoma. The subsequent bone marrow biopsy revealed that the tumor was positive for CD3+, CD5-, CD4-, CD8+, CD56-, CD10-, CD20-, paired box domain 5-, B Cell Leukemia-6-, and multiple myeloma oncogene1-, suggesting the presence of T-cell lymphoma. This case indicates that HPS should be considered when the patient presents with pyrexia, cytopenia, and elevated serum ferritin levels, and the underlying causes must be identified when HPS is confirmed.

In our patient, BMN, rather than hemophagocytosis, was observed in the bone marrow morphology. To date, only a few reports on BMN–LAPS have been documented. It has been demonstrated that bone marrow microenvironment disorder, which is usually caused by malignancy, immune or inflammatory disease, plays an important role in BMN.^[11]^ In our patient, because of the uncontrolled proliferation and activation of EBV-specific cytotoxic T cells and lymphoma cell infiltration, numerous cytokines were secreted in the bone marrow, which could induce bone marrow tissue, stromal cell autophagy, and autolysis, eventually leading to necrosis. The results of bone marrow smears were inconsistent with those of the bone marrow biopsy in this case, which could be related to the different puncture sites.

HPS is usually treated with immunosuppressive therapy, which aims to suppress the hyperinflammatory response and eliminate the activated and infected cells. Based on the 2004 protocol, the patient was treated with etoposide and dexamethasone. However, considering the liver dysfunction, we reduced the dosage of these drugs. Rituximab, a humanized anti-CD20 monoclonal antibody that mainly targets mature B cells, rarely causes severe organ damage. Lim et al^[12]^ found that rituximab is an effective treatment for a number of EBV-mediated conditions, including EBV-induced lymphoproliferative disorders. It has been reported that the application of rituximab in EBV-related HPS can significantly decrease EBV load and limit the inflammatory response in infected B cells.^[13–15]^ Treatment with rituximab had a positive effect on the reduction of EBV load in our patient. Rituximab is much more targeted, specific, less toxic, appears to be well tolerated, and should be included in an optimized treatment regimen.

However, for LAPS, treatment of HPS is insufficient, and a 2-pronged treatment strategy is needed: immunosuppression targeting HPS and cytotoxic chemotherapy targeting lymphoma. Currently, there are no standard guidelines for the treatment of T-cell lymphoma. CHOP and CHOP-like regimens are the recommended first-line treatments, but the total effective rate is poor, and with 5 year survival rate of only 30%.^[16,17]^ Several new anticancer drugs, such as pralatrexate and histone deacetylase inhibitors (chidamide and belinostat), have achieved notable results in the treatment of relapsed and refractory T-cell lymphoma. Hematopoietic stem cell transplantation may provide the opportunity to achieve long-term remission. Fortunately, our patient achieved complete remission owing to CHOPE.

In conclusion, we report a patient with HPS, EBV infection, and BMN associated with T-cell lymphoma. Prompt and accurate diagnosis and identification of the underlying malignancy are important for successful treatment of patients with HPS. CHOPE remains an effective regimen for the treatment of T-cell LAPS. Rituximab can decrease plasma EBV positivity and improve the prognosis. Once LAPS is diagnosed, treatment for lymphoma and HPS is equally important.

## Acknowledgments

The authors thank all the doctors and nurses from the Department of Hematology, Yantai Yuhuangding Hospital, and the team from the Institute of Hematology and Blood Diseases Hospital for their professional assistance.

## Author contributions

**Conceptualization:** Lingling Xu, Xianqi Liu, Yan Wang, Yanming Wang.

**Data curation:** Lingling Xu, Yan Wang.

**Supervision:** Xiaoxia Chu, Liming Chen.

**Writing – original draft:** Lingling Xu.

**Writing – review & editing:** Lingling Xu, Liming Chen.
